# In Vitro Antioxidant and Anti-Neuroinflammatory Effects of *Elsholtzia blanda* (Benth.) Benth.

**DOI:** 10.3390/life15060983

**Published:** 2025-06-19

**Authors:** Yeo Dae Yoon, Krishna K. Shrestha, Seung-Hwa Baek

**Affiliations:** 1 Laboratory Animal Resource Center, Korea Research Institute of Bioscience and Biotechnology, 30 Yeongudanjiro, Cheongju 28116, Republic of Korea; yunyd76@kribb.re.kr; 2 Ethnobotanical Society of Nepal, Kathmandu 44600, Nepal; kkshrestha123@gmail.com; 3 College of Pharmacy, Chungnam National University, 99 Daehak-ro, Yuseong-Gu, Daejeon 34134, Republic of Korea

**Keywords:** *Elsholtzia blanda* (Benth.) Benth. (EBB), antioxidant, anti-neuroinflammatory effect, BV2 microglia cell, proinflammatory cytokine, reactive oxygen species (ROS)

## Abstract

*Elsholtzia blanda* (Benth.) Benth. (EBB) is a traditional plant in Nepal with bioactive properties, including antioxidant, cytotoxic, and antitumor activities. In this study, a methanol EBB extract was tested to evaluate the anti-inflammatory effect against lipopolysaccharide (LPS)-induced microglial (BV2) cells. EBB effectively suppressed LPS-induced nitric oxide production and inhibited the secretion of proinflammatory cytokines such as tumor necrosis factor-alpha (TNF-α) and interleukin (IL)-6. In LPS-stimulated BV2 cells, EBB significantly inhibited inducible nitric oxide synthase (iNOS), TNF-α, IL-6, IL-1β, IL-8, IL-18, and IL-10 mRNA expression in a concentration-dependent manner. In particular, EBB significantly reduced the protein expression of iNOS and cyclooxygenase-2, which were upregulated by LPS. Further, EBB could alleviate the inflammatory response in microglia by suppressing the phosphorylation of mitogen-activated protein kinases. EBB also attenuated LPS-induced reactive oxygen species (ROS) in BV2 cells. In addition, EBB enhanced heme oxygenase-1 (HO-1) protein and mRNA expression. The present results show that an EBB extract could effectively suppress the neuroinflammatory response induced by LPS in BV2 cells. Accordingly, an EBB extract is a promising preventive agent against diseases involving neuroinflammatory responses.

## 1. Introduction

Neuroinflammation is a key mechanism against infectious agents and neuronal injuries in the central nervous system (CNS). However, uncontrolled neuroinflammatory reactions lead to the neuronal damage observed in many neurodegenerative disorders, such as multiple sclerosis, Alzheimer’s (AD), Parkinson’s (PD), Huntington’s disease, and amyotrophic lateral sclerosis (ALS). Microglia are macrophage-like immune cells, comprising approximately 10–20% of the cells in the CNS, and are involved in the initiation of innate immune responses [1]. Although microglial activation is essential for host defense in the brain, abnormal microglia activation can lead to neuroinflammation, a major cause of neurodegenerative diseases [2]. Microglial cells are rapidly activated through interactions with pathogens, and their persistent activation is associated with various neurodegenerative diseases. Since microglia have a limited proliferative capacity, they were newly isolated and used through animal models and primary cultures in each experiment [3]. In particular, the yield of primary microglia (PM) isolated directly from neonatal brain cultures or adult mice has been limited, which has greatly limited their application in neuroscience research [4]. Cultured microglia from mice have been immortalized after infection with a v-raf/v-myc recombinant retrovirus. This immortalized cell line (BV2) exhibits characteristics similar to in vitro macrophages in terms of antigen profile, phagocytosis, and antimicrobial activity [3]. Therefore, many researchers have used the immortalized mouse microglia line BV2 as a PM replacement cell for neuroscience research [3,5,6,7,8,9,10,11,12,13,14,15,16,17].

Activated microglia also release various inflammatory mediators, including tumor necrosis factor-alpha (TNF-α), interleukin (IL)-1β, IL-6, nitric oxide (NO), reactive oxygen species (ROS), and prostaglandin E2 (PGE2), which can be neurotoxic [18]. Microglia activation and the subsequent release of inflammatory mediators, including TNF-α, IL-6, IL-8, IL-10, IL-18, and IL-1β, are reported to increase nerve damage and contribute to neurodegenerative recurrence. Cyclooxygenase-2 (COX-2) and iNOS, which are upregulated in activated microglia, are known to contribute to the etiology of various diseases, including neurodegenerative diseases. Thus, the excessive production of PGE2, an arachidonic acid metabolite produced by COX-2, and NO, a free radical produced by iNOS, activates neuroinflammation and causes brain damage [19].

Lipopolysaccharide (LPS), a major component of endotoxins isolated from Gram-negative bacteria, can be used to induce microglial activation and initiate several key cellular responses involved in the pathogenesis of inflammation [20]. Therefore, LPS-mediated microglia stimulation is widely used to study the underlying mechanisms of neuronal injury mediated through inflammatory and neurotoxic factors [5,6,8,10,11,21]. In addition, hyperactivated microglia stimulated by LPS can increase ROS production, thereby inducing oxidative stress, which further exacerbates the inflammatory response [22]. Additionally, LPS is applied to neural cells and animal models to study neuroinflammation and investigate neurodegenerative diseases such as AD, PD, and ALS [23]. Therefore, blocking microglial activation and the subsequent release of inflammatory mediators is an important tool for delaying the initiation and progression of many neurodegenerative diseases [24].

Heme oxygenase-1 (HO-1) is an antioxidant protein that protects multiple cells from oxidative stress and is crucial for the cellular stress response. HO exists in three types, HO-1, -2, and -3, among which HO-1 protects cells from free radicals and NO and regulates inflammatory responses [25]. HO-1 induction in neurons and microglia is associated with the nuclear transcription factor-E2-related factor 2 and is in a cell defense system that prevents the oxidative damage caused by inflammation [26]. Reportedly, cerebellar granule cells from transgenic mice with neurons engineered to overexpress HO-1 are resistant to H_2_O_2_-mediated oxidative damage in vitro [27]. Moreover, phytochemicals such as quercetin, morin, ginsenoside Rg18, xanthohumol, resveratrol, totarol, curcumin, caffeic acid phenethyl ester, and epigallocatechin-3-gallate alleviate neuronal damage by upregulating HO-1 expression in cells subjected to oxidative stress [28].

*Elsholtzia blanda* (Benth.) Benth. (EBB) is a perennial plant of the Lamiaceae family widely distributed in Nepal, Malaysia, India, Vietnam, Korea, Bangladesh, and China [29]. Traditionally, extracts of *Elsholtzia* have been used in folk medicine in China to treat indigestion, nephritis, rheumatoid arthritis, colds, pharyngitis, and headaches [30]. Ling et al. reported that the total flavones from EBB could improve myocardial function and protect the heart from ischemic damage caused by coronary artery occlusion in beagle dogs [31]. Devi et al. showed that the methanol extract of EBB could exhibit nephroprotective effects through antioxidant activity in albino rat model [32]. Ling et al. reported that the total flavones of EBB can reduce acute myocardial infarction by inhibiting myocardial apoptosis through Bcl-2 family regulation [33]. Additionally, various phenolic compounds were identified in the EBB extract [34,35,36]. However, despite studies on the antimicrobial [37], anti-ischemic [33], and anti-inflammatory properties [38] of EBB, no research has focused on its anti-neuroinflammatory effect in microglia. Thus, this study aimed to investigate the anti-neuroinflammatory and antioxidant effects of EBB on BV2 microglial cells stimulated with LPS and to investigate the underlying mechanisms.

## 2. Materials and Methods

### 2.1. Materials

LPS (from an *Escherichia coli* strain), dimethyl sulfoxide (DMSO, CAS. No. 37-68-5, ≥99.7%), sulfanilamide (CAS. No. 63-74-1, ≥98%), naphthyl ethylenediamine dihydrochloride (CAS. No. 1465-25-4, ≥98%), N-acetyl-L-cysteine (NAC, CAS. No. 616-91-1, ≥99%), hispidulin (CAS. No. 1447-88-7, ≥98%), and phosphoric acid (CAS. No. 7664-38-2, ≥99%) were obtained from Sigma (St. Louis, MO, USA). Dulbecco’s Modified Eagle’s Medium (DMEM), penicillin (100 IU/mL)-streptomycin (100 μg/mL), fetal bovine serum (FBS), and trypsin-ethylenediaminetetraacetic acid (EDTA) were purchased from Gibco (Grand Island, NY, USA). Primary antibodies against cyclooxygenase-2 (COX-2), inducible nitric oxide synthase (iNOS), p-extracellular signal-regulated kinase1/2 (ERK1/2), ERK1/2, p-p38, p38, p-stress-activated protein kinases/Jun-amino-terminal kinases (SAPKs/JNKs), SAPKs/JNKs, and β-actin were purchased from Cell Signaling Technology (Danvers, MA, USA). Horseradish peroxidase (HRP)-conjugated antirabbit IgG, HRP-conjugated antimouse IgG, and heme oxygenase-1 (HO-1) were obtained from Santa Cruz Biotechnology (Santa Cruz, CA, USA). Cell-permeant 2′,7′-dichlorodihydrofluorescein diacetate (H_2_DCFDA) was purchased from Invitrogen (Waltham, CA, USA).

### 2.2. Plant Material and Identification

*Elsholtzia blanda* (Benth.) Benth. (EBB) was collected from Jukeghari, Phulkharka-3, Dhading, Nepal. The plant samples were collected and taxonomically identified by Krishna K. Shrestha at the Ethnobotanical Society of Nepal, Kathmandu, Nepal. A voucher specimen (EK 0597) was deposited in the herbarium of the Korea Research Institute of Bioscience and Biotechnology (KRIBB), Daejeon, Korea, for future reference. The EBB aerial extracts were obtained from the KRIBB (125, Gwahak-ro, Yuseong-gu, Daejeon 34141, Republic of Korea, FBM230-064). EBB young branches (100 g), which were dried and ground, were treated with 750 mL of methanol (HPLC grade) at room temperature and extracted for one day at room temperature. The sample solution was placed in a sonicator (Digital Ultrasonic Cleaner LMUC series; Maharashtra, India) and underwent continuous cycles of sonication at 40 kHz for 5 cycles of 15 min each at 45 °C. This process was carried out for three days for complete extraction. Filtration was performed under reduced pressure (DYN AIR^®^, noiseless air compressor; FuJian, China) using filter paper (Whatmann filter paper No.1; Millipore Cor., MA, USA). The filtrate was evaporated under a rotary vacuum evaporator (IKA, RV 10, Staufen, Germany) at 45 °C. The yield of methanol extract was 2 g (2%). EBB was dissolved in DMSO, prepared to a 10 mg/mL stock, and stored at −20 °C. Therefore, the vehicle (VH) was treated with 0.01% DMSO.

### 2.3. Cell Culture and Viability

BV2 microglia were purchased from AcceGen (Fairfield, NJ, USA). Cells were cultured at 37 °C in DMEM supplemented with 10% FBS and 1% penicillin-streptomycin, in a humidified 5% CO_2_ atmosphere. Cells were pretreated with EBB for 1 h before stimulation with 0.2 μg/mL LPS for the indicated time. For cell viability, after 24 h of EBB treatment (2.5–10 μg/mL) with/without LPS, the incubation medium was discarded, and 10% cell counting kit 8 reagent (Dojindo Molecular Technologies, Inc., Kumamoto, Japan) in DMEM was added to each well. After further incubation at 37 °C for 30 min, the absorbance of each well was measured at 450 nm using a microplate reader (Bioteck; Agilent, Santa Clara, CA, USA).

### 2.4. NO Measurements

The nitrite concentration in the culture supernatants was measured by the Griess method [39]. Briefly, BV2 cells (5 × 10^5^ cells/mL) were seeded into 96-well plates. After seeding, the cells were pretreated with EBB at different concentrations for 1 h, followed by incubation with LPS for 24 h. Then, the supernatant was collected, and 90 μL of it was added into a 96-well plate with 90 μL of Griess reagent (1% [*w*/*v*] sulfanilamide in 5% [*v*/*v*] phosphoric acid and 0.1% [*w*/*v*] naphthylethylenediamine-HCl). After 10 min of incubation at room temperature in the dark, the absorbance at 540 nm was measured using a microplate reader. The nitrite concentration in the samples was calculated from a sodium nitrite standard curve. In a previous study, hispidulin (HPD), which was confirmed to have NO inhibitory activity in BV2 cells, was used as a positive control to verify that the detection system was functioning properly in the NO assay [15].

### 2.5. Intracellular ROS Assay

ROS levels within cells were measured by assessing H_2_DCFDA oxidation [40]. BV2 cells were plated at a density of 4 × 10^5^ cells/mL in a 96-well black–clear plate, pretreated with EBB for 1 h, and stimulated with LPS for 24 h. Then, the cells were washed with phosphate-buffered saline (PBS) and treated with 10 μM H_2_DCF-DA reagent. After incubation in the dark for 30 min at 37 °C, cells were washed twice with PBS. Next, the fluorescence was measured using a microplate reader at 485 nm excitation and at 535 nm emission wavelengths. The fluorescence intensities were calculated using Gen5 software (Bioteck; Agilent, Santa Clara, CA, USA). Cells were visualized using the CELENA^®^ S Digital Imaging System (Logos Biosystems, Anyang, Gyeonggido, Republic of Korea). Similarly, cells cultured in 6-well plates were subjected to the same treatments and measured using flow cytometry (FACS). The FACS data were measured with a BD FACSCalibur (BD Biosciences, San Jose, CA, USA) at 488 nm excitation and 525 nm emission wavelengths. The data were analyzed using FlowJo software (Version 10; Tree Star, San Carlos, CA, USA).

### 2.6. IL-6 and TNF-α Production

IL-6 and TNF-α concentrations in the culture supernatant were determined using enzyme-linked immunosorbent assay (ELISA) kits, as described previously [41]. All the ELISA kits were purchased from R&D Systems (Rochester, MN, USA).

### 2.7. Western Blot Analysis

Protein extraction from the cells was performed using a radioimmunoprecipitation assay buffer (50 mM Tris-Cl [pH 8.0], 5 mM EDTA, 150 mM NaCl, 1% NP-40, 0.1% SDS, and 1 mM phenylmethylsulfonyl fluoride), as described previously [42]. The cell lysates were centrifuged at 13,000× *g* for 20 min at 4 °C to collect the supernatants. The bicinchoninic acid assay (ThermoFisher; Waltham, MA, USA) was conducted to measure protein concentrations. Proteins (25 μg/well) were separated using 10% sodium dodecyl sulfate–polyacrylamide gel electrophoresis and transferred to polyvinylidene fluoride membranes (Millipore, Bedford, MA, USA), blocked with 5% skim milk at 4 °C for 1 h, and incubated with specific primary antibodies at 4 °C overnight. Then, these membranes were incubated with the corresponding HRP-conjugated secondary antibody at room temperature for 2 h. The expressed protein bands were detected using an enhanced chemiluminescence solution (ThermoFisher; Waltham, MA, USA). The band intensity was measured with a ChemiDoc MP Imaging System (Bio-rad; Hercules, CA, USA). All the Western blot analyses were performed in triplicate.

### 2.8. Total RNA Extraction

The total RNA was extracted using TRIzol (Invitrogens; Waltham, MA, USA) according to manufacturer’s instructions [43]. Briefly, 200 μL of chloroform was added, and tubes with the lysis mixture were gently inverted for 5 min. Then, the mixture was centrifuged at 12,000× *g* for 20 min at 4 °C, and the clear upper solution was placed into a new tube, before adding 500 μL of isopropanol. Then, the tubes were inverted on ice for 15 min, followed by centrifugation at 12,000× *g* for 20 min at 4 °C, and the isopropanol was decanted before incubation. Next, ice-cold 70% ethanol was added to the RNA pellets for gentle washing. After centrifuging as indicated above, for 10 min, the ethanol was removed, and the RNA pellets were dried at room temperature for 5–10 min before reconstitution in 20 μL diethyl pyrocarbonate-treated water. The RNA quality was assessed using a nanodrop spectrophotometer (Bioteck; Agilent, Santa Clara, CA, USA). RNA purity allows 1.8–2.0 values for the A_260_/A_280_ ratio.

### 2.9. Real-Time Reverse Transcription–Polymerase Chain Reaction

Real-time reverse transcription–polymerase chain reaction (RT-PCR) was performed in 20 µL of solution containing 10 µM primers and the total RNA in One-Step TB RT-PCR buffer and enzyme mix (One-Step TB PrimeScript RT-PCR Kit II, Takara Bio Inc., Otsu, Shiga, Japan) [44]. The reverse transcription process was carried out for 5 min at 42 °C, followed by 10 s at 95 °C. The PCR reaction comprised 50 cycles at 95 °C for 5 s, followed by 34 s at 60 °C. Immediately thereafter, a melting curve analysis was performed to confirm the specificity of each PCR product. All the reactions were run in triplicate, and the relative expression levels were determined using the 2^−∆∆CT^ method normalized to the expression levels of glyceraldehyde-3-phosphate dehydrogenase (GAPDH). The primer sequences used in this study are listed in Table 1.

### 2.10. Statistical Analysis

All the statistical analyses were performed using Prism software (Version 5; GraphPad Inc., San Diego, CA, USA). Differences between groups were assessed using one-way ANOVA followed by Tukey’s post hoc test. All numerical data are presented as the mean ± standard deviation (SD) of ≥3 repetitions, with differences considered significant at *p* < 0.05.

## 3. Results

### 3.1. Effects of EBB on NO Production and Cell Viability in LPS-Stimulated BV2 Cells

**Figure 1:**
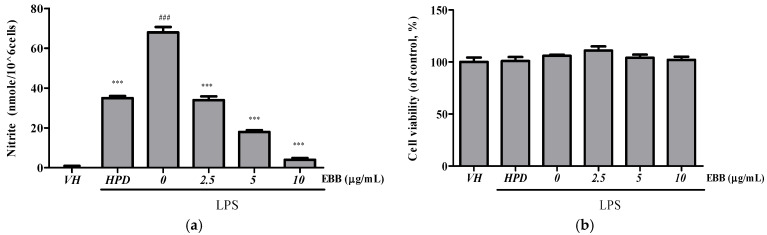
Effect of EBB on NO production in LPS-stimulated BV2 cells. BV2 cells were pretreated with various concentrations of EBB for 1 h, followed by incubation with LPS (0.2 µg/mL) for 24 h. Then, NO levels in the supernatant were determined using the Griess reagent (**a**). Cell viability was determined using the CCK-8 assay (**b**). Hispidulin (HPD, 10 μM) is the positive control. Each column represents mean ± SEM (n = 5). ^###^ *p* < 0.001 vs. VH, *** *p* < 0.001 vs. LPS-treated group.

The viability of BV2 cells at 2.5 and 10 μg/mL EEB was determined prior to NO production analyses (Figure 1a). Nontoxicity was confirmed up to 10 μg/mL EEB (Figure 1b). To investigate the anti-inflammatory properties of the extract, the effect on LPS-induced NO production was determined by measuring the NO levels in BV-2 cells after 24 h of treatment. The NO level significantly increased in LPS-stimulated control cells compared with that in unstimulated control cells, and pretreatment with EBB (10 μg/mL) significantly inhibited LPS-induced NO production by up to 94%.

### 3.2. Effects of EBB on Cytokine Production and mRNA Expression in LPS-Stimulated BV2 Cells

**Figure 2:**
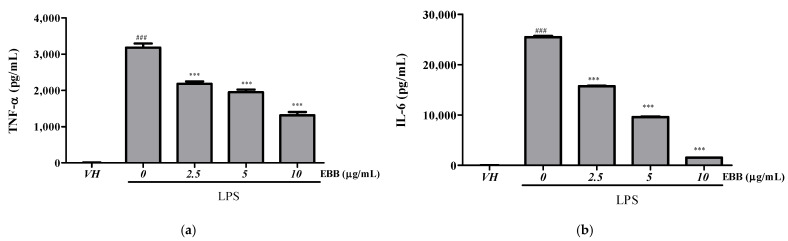
Effects of EBB on LPS-induced production of TNF-α (**a**) and IL-6 (**b**) in BV2 cells. Cells were pretreated with different concentrations of EBB for 1 h before treatment with 0.2 μg/mL LPS. After incubation for 18 h, the IL-6 and TNF-α production was measured by ELISA kits. Each column represents mean ± SEM (n = 5). ^###^ *p* < 0.001 vs. VH, *** *p* < 0.001 vs. LPS-treated group.

**Figure 3:**
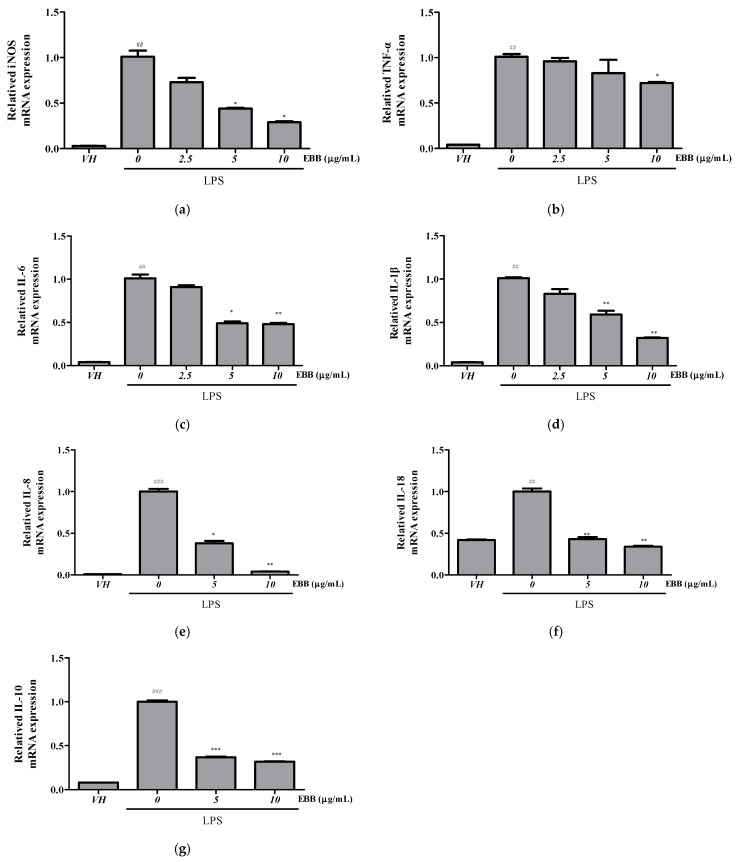
Effect of EBB on the *iNOS*, *TNF-α*, *IL-6*, *IL-1β*, *IL-18*, *IL-8*, and *IL-10* gene expression in BV2 cells. Cells were pretreated with EBB for 1 h prior to LPS stimulation. After 6 h of LPS stimulation, total RNAs were isolated using a Trizol reagent kit. The *iNOS* (**a**), *TNF-α* (**b**), *IL-1 β* (**c**), *IL-6* (**d**), *IL-8* (**e**), *IL-18* (**f**), and *IL-10* (**g**) mRNA levels were accessed by real-time RT-PCR. The mRNA levels were determined by the Ct-value method and normalized by the housekeeping gene GAPDH. Each column represents mean ± SEM (n = 3). ^##^ *p* < 0.01, ^###^ *p* < 0.001 vs. VH, * *p* < 0.05, ** *p* < 0.01, *** *p* < 0.001 vs. LPS-treated group.

Compared with the TNF-α levels in control cells, the levels in BV2 cell culture media significantly increased after LPS treatment. However, after pretreatment with EBB, the TNF-α level significantly decreased in a concentration-dependent manner compared with that in cells treated only with LPS (Figure 2a). Similarly, the IL-6 levels significantly decreased in the EBB-treated group compared with those in the LPS-treated group (Figure 2b). RT-PCR indicated overexpression of *iNOS*, *TNF-α*, *IL-1β*, *IL-6*, *IL-8*, *IL-18*, *IL-10* mRNA in LPS-induced cells. However, EBB treatment significantly downregulated the LPS-induced increased *iNOS*, *TNF-α*, *IL-1β*, *IL-6*, *IL-8*, *IL-18*, and *IL-10* mRNA expression levels (Figure 3a–g).

### 3.3. Effects of EBB on Inflammatory-Related Proteins of LPS-Stimulated BV2 Cells

**Figure 4:**
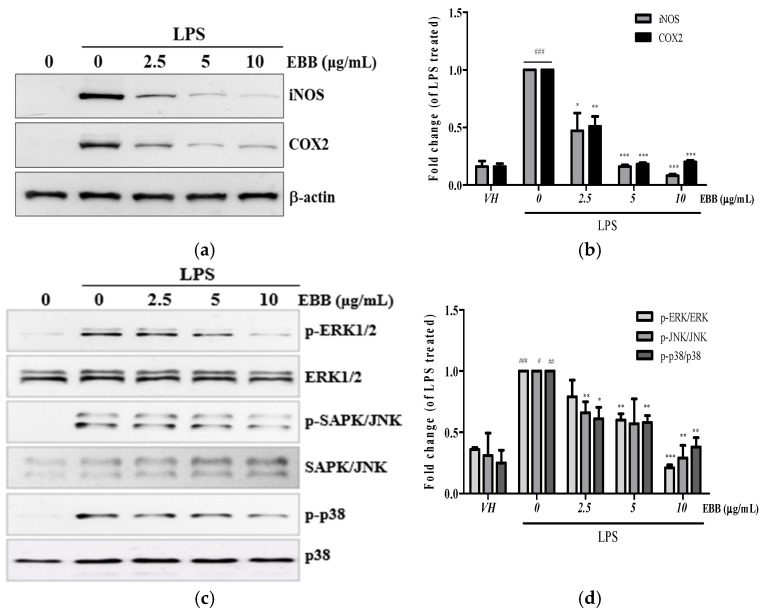
Effects of EBB on LPS-induced protein expression of iNOS, COX-2, and mitogen-activated protein kinase (MAPK) phosphorylation in BV2 cells. Cells were pretreated with different concentrations of EBB for 1 h before treatment with 0.2 μg/mL LPS. After incubation for 24 h, the iNOS and COX-2 protein levels were measured by Western blotting (**a**,**b**). After incubation for 15 min, the MAPK phosphorylation and non-phosphorylation levels were analyzed (**c**,**d**). The band intensity was analyzed by ImageJ program (Version 1.54p). Each column represents mean ± SEM (n = 3). ^#^ *p* < 0.05, ^##^ *p* < 0.01, ^###^ *p* < 0.001 vs. VH, * *p* < 0.05, ** *p* < 0.01, *** *p* < 0.001 vs. LPS-treated group.

Next, we examined the iNOS and COX-2 protein expression levels by Western blot. As shown in Figure 4a, the increased iNOS and COX-2 protein expression induced by LPS was inhibited by EBB in a dose-dependent manner. Mitogen-activated protein kinase (MAPK) signaling is a key regulator of inflammatory cytokines in LPS-stimulated microglia. Therefore, to determine whether EBB inhibits the production of inflammatory cytokines via the MAPK signaling pathway, the ERK1/2, SAPKs/JNKs, and p38 phosphorylation levels were analyzed by Western blot in LPS-treated BV2 microglia. Stimulation with LPS significantly increased ERK1/2, SAPKs/JNKs, and p38 phosphorylation. However, pretreatment with EBB (10 μg/mL) inhibited ERK1/2, SAPKs/JNKs, and p38 phosphorylation (Figure 4b). These results suggest that EBB has an anti-inflammatory effect through the phosphorylation pathway of ERK1/2, SAPKs/JNKs, and p38 in LPS-induced inflammatory cytokine production (See Supplementary Materials).

### 3.4. Effects of EBB on ROS Production of LPS-Treated Microglial Cells

**Figure 5:**
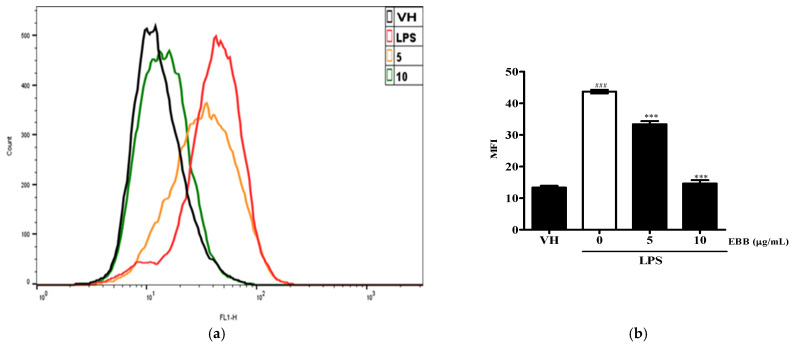

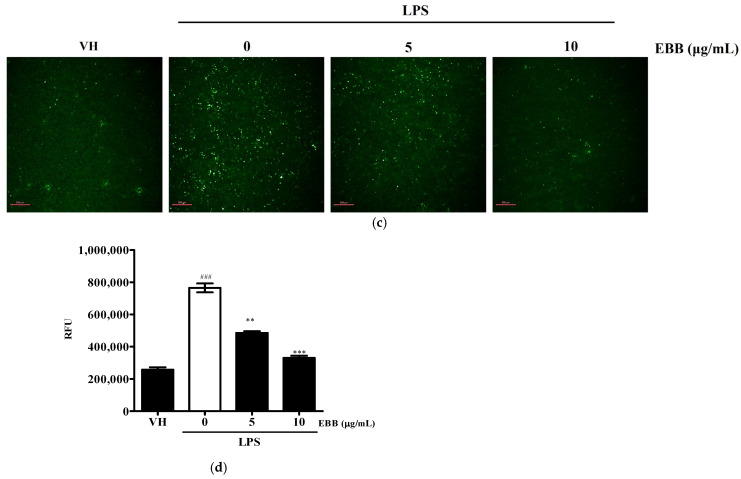
Effects of EBB on LPS-induced ROS production. BV2 cells were pretreated with 5 and 10 μg/mL EBB for 1 h before treatment with 0.2 μg/mL LPS for 18 h. Cells were incubated with 10 μM 2′,7′-dichlorofluorescein diacetate (DCFH-DA) for 30 min at 37 °C. Cells were harvested, and the dichlorofluorescein (DCF) fluorescence levels were immediately analyzed by flow cytometry (**a**). DCF fluorescence intensities were determined from the same numbers of cells in a randomly selected area. The mean relative ROS level is presented in a bar graph. (**b**). The intracellular levels of ROS were then determined by fluorescence microscopy (**c**) and microplate reader (**d**). The values are presented as means ± SD of three independent experiments. ** *p* < 0.01, *** *p* < 0.001 compared with the LPS-treated group. ^###^ *p* < 0.001 vs. VH; scale bar, 200 μm.

To determine whether the anti-neuroinflammatory effect of EBB was caused by a reduction in ROS generation, the intracellular ROS production in BV2 cells was measured by DCFH-DA. Flow cytometry analysis showed that intracellular ROS accumulation occurred at 30 min, and that ROS production significantly decreased with increasing EBB concentration(Figure 5a,b). Next, to visualize the inhibitory effect of intracellular ROS production by EBB, the cells were identified using fluorescence microscopy. As shown in Figure 5c, it was observed that LPS-induced ROS decreased in a concentration-dependent manner upon EBB treatment. In addition, as a result of measuring the degree of fluorescence using a microplate reader to quantify this, the ROS fluorescence levels increased significantly in LPS-treated cells compared with control cells, and after EBB treatment, ROS production was significantly suppressed compared with that in the LPS-treated cell group (Figure 5d).

### 3.5. Anti-Inflammatory Effects of EBB Are Mediated by the HO-1 Signaling Pathway

**Figure 6:**
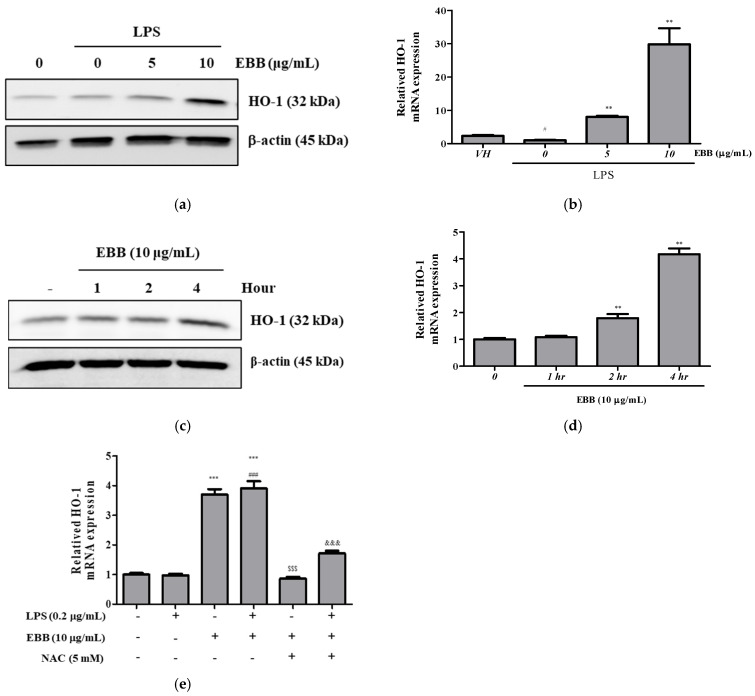
Effects of ROS scavenger on HO-1 expression in BV2 cells. BV2 cells were pretreated with EBB for 1 h before treatment with 0.2 μg/mL LPS for 24 h. The protein expression of HO-1 was determined by Western blotting assay (**a**). The mRNA expression of HO-1 was determined by real-time RT-PCR (**b**). BV2 cells were treated with 10 μg/mL EBB for 0, 1, 2, and 4 h, and then HO-1 protein expression levels were measured (**c**). The mRNA expression of HO-1 was determined by real-time RT-PCR (**d**). *HO-1* mRNA expression levels in BV2 cells pretreated with EBB and 5 mM NAC and then stimulated with 0.2 µg/mL LPS for 2 h (**e**). The values are presented as means ± SD of three independent experiments. ^#^ *p* < 0.05, ^###^ *p* < 0.001 compared to the VH; ** *p* < 0.01, *** *p* < 0.001 vs. LPS treated cells; ^$$$^ *p* < 0.001 vs. EBB-treated cells; ^&&&^ *p* < 0.001 vs. LPS + EBB-treated cells.

The HO-1 signaling pathway, which is essential for oxidative stress, is involved in microglial regulation. The expression of HO-1 at the protein and gene levels in LPS-induced BV2 cells showed no significant difference from that in the control group, whereas after EBB treatment, the protein and gene expressions were significantly increased. HO-1 was effectively increased by EBB pretreatment, even during LPS stimulation (Figure 6a,b). Next, we examined the protein and gene levels of HO-1 after EBB treatment alone in BV2 cells. EBB increased the HO-1 protein and gene expression levels, with the maximum increase detected after 4 h of treatment (Figure 6c,d). Notably, the HO-1 protein and gene were upregulated in a time-dependent manner after EBB treatment. Next, to measure the HO-1 mRNA expression level in cells treated with NAC, which is a ROS scavenger, BV2 cells were cotreated with EBB and 5 mM NAC for 4 h. The EBB-induced HO-1 mRNA expression levels were reduced after the NAC cotreatment (Figure 6e). Pretreatment of BV2 cells with EBB and 5 mM NAC for 2 h followed by stimulation with LPS for 4 h revealed the absence of an effect of the LPS treatment on the EBB-upregulated HO-1 mRNA expression levels. However, after treatment with NAC, the HO-1 mRNA levels were reduced in cells treated with LPS and EBB, as well as in those treated with EBB alone (Figure 6e).

## 4. Discussion

Microglia are specialized immune cells in the CNS that play an important role in maintaining brain homeostasis and preventing neurodegenerative diseases [18]. In a healthy state, the microglia play a role in pathogen clearance and tissue repair in the event of injury or infection, while dysregulation can lead to neuroinflammation or nerve damage [45]. Microglia release inflammatory molecules such as cytokines, chemokines, and free oxygen, which can cause multiple chronic inflammations, destroy healthy neurons, and increase the progression of neurodegenerative diseases [2]. Thus, many therapeutic development strategies have been based on the understanding of the activation and functional regulation of microglia in neurodegenerative diseases [46,47]. Because inflammatory bacterial LPS induces microglial activation via the toll-like receptor (TLR)-4 pathway to cause neurological diseases [48], the effect of inhibiting LPS-induced microglial inflammatory responses is widely used to screen active substances for neurodegenerative diseases. Therefore, our study used LPS-induced BV2 microglia to investigate the anti-inflammatory activity of EBB in neurodegeneration.

Our results showed that EBB pretreatment suppressed NO production (Figure 1a) and decreased TNF-α and IL-6 cytokine secretion in LPS-activated mouse microglia (Figure 2a,b). Simultaneously, we found that EBB inhibited iNOS and the COX-2 protein expression (Figure 4a). The release of proinflammatory cytokines (TNF-α and IL-6), which cross the blood–brain barrier, congregates leukocytes and amplifies the inflammatory response, which in turn induces neuroinflammatory processes [49]. In addition, high concentrations of COX-2 can increase prostaglandins, which are inflammatory mediators, and cause neurotoxicity [45]. In mammalian cells, three nitric oxide synthases (NOSs), namely, neural NOS (nNOS), endothelial NOS (eNOS), and iNOS, are present. The three NOS isoforms are regulated differently, of which iNOS is expressed in response to inflammatory stimuli. In particular, the excessive expression of iNOS causes brain cell tissue destruction due to the production of high NO and peroxynitrate, which causes several degenerative diseases [50,51]. Taken together, our results suggest that EBB may play a role in attenuating the neurotoxic effects of LPS by inhibiting iNOS protein expression and reducing the production of the neurotoxic mediator NO and the inflammatory cytokines TNF-α and IL-6.

In microglia, LPS can induce MAPK phosphorylation to activate and release inflammatory cytokines, leading to neuroinflammation [52]. MAPKs mainly consist of three well-known subfamilies, including ERK1/2, SAPKs/JNKs, and p38 MAPK, and MAPK phosphorylation activated by LPS induces the expression of inflammatory mediators, which induces various neurological diseases [53]. In this study, the Western blotting analysis results showed that LPS treatment increased ERK1/2, SAPKs/JNKs, and p38 MAPK phosphorylation levels in BV2 microglia, an effect inhibited by EBB treatment (Figure 4c). Taken together, these results suggest that LPS-induced MAPK phosphorylation may play an important role in increasing TNF-α and IL-6 production through microglial activation and that EBB can inhibit inflammatory mediators by inhibiting MAPK phosphorylation.

Along with inflammatory damage, oxidative stress is another major cause of damage to the nervous system. ROS serve as central regulators of inflammatory signaling; particularly excessive production of ROS and reactive nitrogen species can destroy redox homeostasis, resulting in oxidative stress, which is known to damage neurons and tissues and affect normal signaling pathways [54]. Previous studies reported that the increased ROS production in microglia caused by LPS is directly related to the inflammatory response, while the inhibition of the inflammatory response is related to ROS suppression [16,17,55]. Furthermore, a recent study reported that natural plant extracts have anti-inflammatory and antioxidant effects by blocking microglia activation [7,12,13,56,57,58,59]. We observed that EBB treatment removed LPS-induced ROS, showing a protective effect in BV2 cells, suggesting that EBB may be effective in maintaining cellular redox balance and may inhibit the development or progression of oxidation-related neurodegenerative diseases.

HO-1 can inhibit nitrite production as a member of the stress protein superfamily, is also known as heat-shock protein 32, and plays an important role in regulating biological oxidative stress systems [53]. Therefore, we evaluated the regulatory effect of HO-1 on the inflammatory response to EBB in LPS-activated BV2 microglia. In this study, HO-1 was effectively increased by EBB pretreatment even during LPS stimulation (Figure 6a,b), and HO-1 mRNA expression and protein levels were increased through EBB-alone treatment, with maximum induction at 4 h (Figure 6c,d). This suggests that the neuroprotective effect of EBB is potentially mediated by the expression of HO-1. In addition, the increase in the expression levels of the HO-1 mRNA afforded by EBB decreased after NAC treatment (Figure 6e). Therefore, the HO-1 induction by EBB suggests that it is associated with ROS production. Antioxidant proteins, such as HO-1, are known to play a critical role in suppressing neuroinflammation and the resulting nerve cell loss [60]. In several in vitro studies, the activation of HO-1 has confirmed neuroprotective effects against brain diseases by relieving neuroinflammation by inhibiting the production of NADPH oxidase and reactive quinones, which are key enzymes responsible for the release of reactive oxygen in microglia [61,62,63,64]. Transcriptional upregulation of the HO-1 gene occurs after the exposure of cells to a wide range of stimuli such as hydrogen peroxide (H_2_O_2_), ultraviolet rays, cytokines, and bacterial endotoxins, which directly or indirectly produce intracellular ROS. Therefore, ROS could play a secondary messenger role in the upregulation of HO-1 expression [65]. ROS removal using NAC has been shown to affect the HO-1 expression of EBB. However, further studies are needed in the future as it is necessary to confirm whether HO-1 induction by EBB occurs upstream or downstream of ROS changes. HO-1 is known to be regulated by nuclear factor erythrocyte 2-related factor 2 (Nrf2) upon the occurrence of oxidative stress [66]. In the steady state, Nrf2 exists in the form of binding to Kelch-like ECH-related protein 1 (Keap1) within the cytoplasm, and when activated by stimulation, it separates from Keap1, moves to the nucleus, and then binds to antioxidant response elements to activate HO-1 gene transcription. Furthermore, it is known that the nuclear accumulation of Nrf2 occurs through various protein kinase pathways, such as the MAPK pathway. Balogun et al. reported that curcumin upregulates HO-1 via the p38 pathway in porcine renal epithelial proximal tubule cells [67]. Kietzmann et al. reported that sodium arsenite has been shown to regulate HO-1 expression by activating the JNK and the Ras pathway but not the ERK pathway in rat hepatocytes [68]. Martin et al. indicate that carnosol activates HO-1 expression by increasing Nrf2 protein levels in a PI3K/Akt-dependent manner [69]. Foresti et al. identified four HO-1 activators (cobalt protoporphyrin, carnosol, curcumin, and dimelthyl fumarate) that can modulate inflammation via the Nrf2/HO-1 pathway in BV2 microglia [14]. Therefore, further mechanism studies are needed to confirm the direct association between ROS inhibition and the anti-inflammatory efficacy of EBB.

Immortalized mouse microglia line BV2 has been frequently used as a PM replacement cell, but it does not capture all the features of microglia. However, transcriptome and proteome analyses after lipopolysaccharide stimulation revealed that the response pattern of BV2 was very similar to that of PM and showed normal NO production regulation and functional response to IFN-γ [3]. Therefore, BV2 cells have been used by many researchers as effective replacement cells for PM in several neurosciences, including complex inter-cell interaction studies, in their studies. However, to confirm the more definitive neuroprotective effect of EBB, in vivo experiments such as mouse experiments are required.

The pharmacological effects of plant extracts are related to the overall therapeutic effects of various plant compounds contained in the extract [70]. In addition, EBB is a crude extract, so its safety profile has not yet been studied. However, several studies have found a variety of phenolic compounds in EBB extracts [34,35,36]. Therefore, further studies of a single compound derived from EBB are essential to obtain a more comprehensive understanding of the pharmacological effects of EBB. Furthermore, further studies on whether EBB-active components reach the central nervous system through blood–brain barrier permeability studies, together with acute and chronic toxicity assessments, for extensive application to neuroprotective effects will be needed.

## 5. Conclusions

In conclusion, the present study showed that EBB exerted potent anti-neuroinflammatory effects on microglial activation by LPS, possibly through the inhibit NO, TNF-α, and IL-16 production in LPS-stimulated BV2 microglia by MAPK phosphorylation inhibition. Furthermore, we confirmed the activation of HO-1 expression by EBB in BV-2 cells, demonstrating that HO-1 is involved in mediating antioxidant and anti-neuroinflammatory effects. Therefore, blocking the excessive inflammatory response in microglia suggests that it is an important tool to delay the induction and progression of numerous brain diseases. However, although the current findings may provide a partial understanding of the mechanisms of the anti-neuroinflammatory effects of EBB, further studies are needed to assess the additional role of EBB in various oxidative stress and neuroinflammatory diseases.

## Figures and Tables

**Table 1:** Primer sequence list.

Gene	Primer Sequence	Accession No.
mouse iNOS	Forward 5′-ATGGACCAGTATAAGGCAAGC-3′ Reverse 5′-GCTCTGGATGAGCCTATATTG-3′	BC062378
mouse TNF-α	Forward 5′-GGTGCCTATGTCTCAGCCTCTT-3′ Reverse 5′-GCCATAGAACTGATGAGAGGGAG-3′	BC117057
mouse IL-6	Forward 5′-TACCACTTCACAAGTCGGAGGC-3′ Reverse 5′-CTGCAAGTGCATCATCGTTGTTC-3′	BC132458
mouse IL-1β	Forward 5′-TGGACCTTCCAGGATGAGGACA -3′ Reverse 5′- GTTCATCTCGGAGCCTGTAGTG -3′	BC011437
mouse IL-8	Forward 5′-CTCTATTCTGCCAGATGCTGTCC-3′ Reverse 5′-ACAAGGCTCAGCAGAGTCACCA-3′	BC051677
mouse IL-18	Forward 5′-GACAGCCTGTGTTCGAGGATATG-3′ Reverse 5′-TGTTCTTACAGGAGAGGGTAGAC-3′	NM_008360
mouse IL-10	Forward 5′-CGGGAAGACAATAACTGCACCC-3′ Reverse 5′-CGGTTAGCAGTATGTTGTCCAGC-3′	NM_010548
mouse HO-1	Forward 5′-TTACCTTCCCGAACATCGAC-3′ Reverse 5′-GCATAAATTCCCACTGCCAC-3′	BC010757
mouse GAPDH	Forward 5′-GCGAGACCCCACTAACATCA-3′ Reverse 5′-GAGTTGGGATAGGGCCTCTCTT-3′	GU214026

## Data Availability

Data are contained within the article.

## Supplementary Materials

The following supporting information can be downloaded at: https://www.mdpi.com/article/10.3390/life15060983/s1, Figure S1: Original figure of western blot for Figure 4a. The concentration of 20 μg/mL was excluded from the results because it was cytotoxic; Figure S2: Original figure of western blot for Figure 4c. The concentration of 20 μg/mL was excluded from the results because it was cytotoxic; Figure S3: Original figure of western blot for Figure 4c. If you adjust the contrast, you can see the marker band. After confirming with that, we checked the band at the desired location; Figure S4: Original figure of western blot t for Figure 6a. If you adjust the contrast, you can see the marker band. After confirming with that, we checked the band at the desired location; Figure S5: Time-course analysis of ERK1/2, SAPK/JNK, and p38/MAPK phosphorylation in response to LPS. BV2 cells were treated with LPS (0.2 μg/mL). Equal amounts of cell lysates, harvested at the indicated time points, were analyzed for ERK1/2, SAPK/JNK, and p38/MAPK phosphorylation by western blot analyses.
