# Supplementary material for: In Vitro Antioxidant and Anti-Neuroinflammatory Effects of Elsholtzia blanda (Benth.) Benth
Source: Life (Basel). 2025 Jun 19;15(6):983. doi: 10.3390/life15060983 (PMC12193901; doi:10.3390/life15060983)
Supplement: Supplementary file 1 [file life-15-00983-s001.zip › life-3664905-supplementary.pdf]

In Vitro Antioxidant and Antineuroinflammatory Effects of *Elsholtzia blanda* Benth

SUPPLEMENTAL MATERIAL

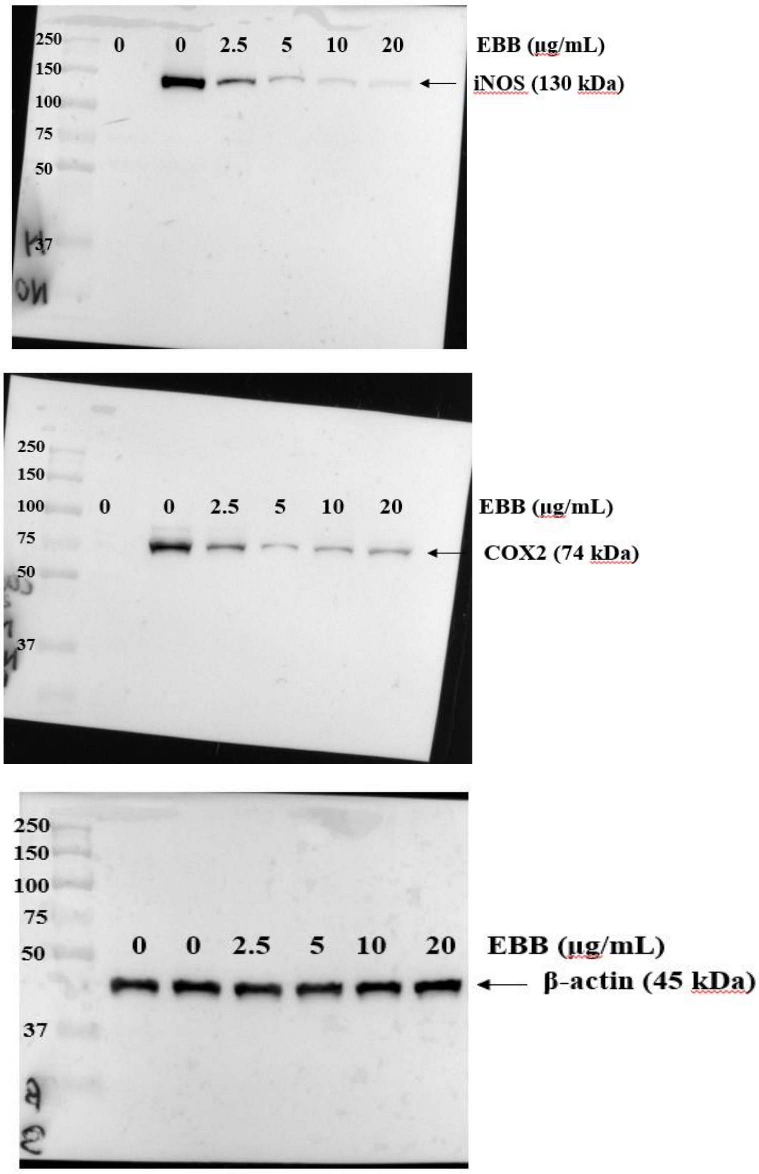

Supplementary Figures S1. Original figure of western blot for Figure 4a. The concentration of 20 µg/mL was excluded from the results because it was cytotoxic.

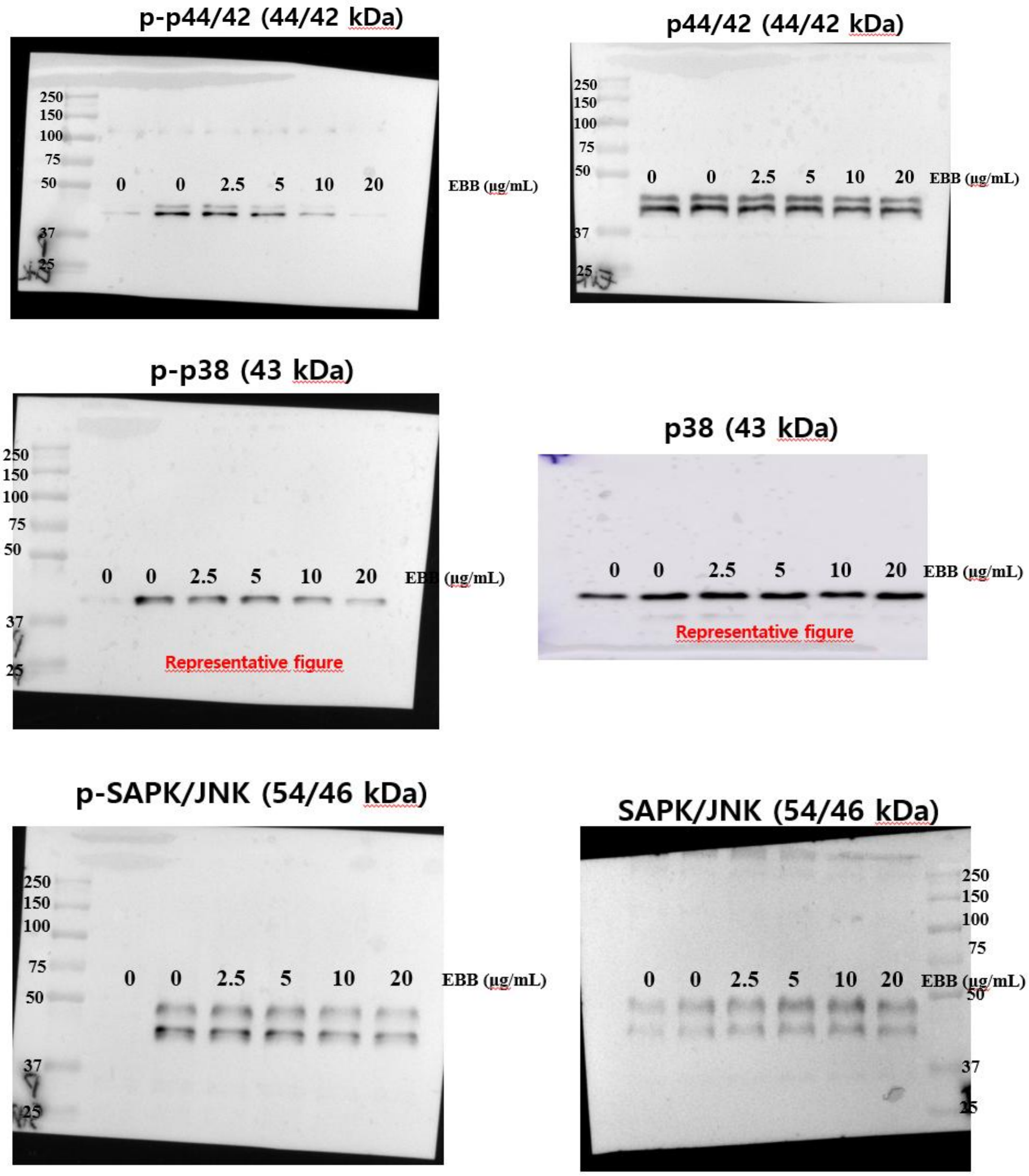

Supplementary Figures S2. Original figure of western blot for Figure 4c. The concentration of 20 μg/mL was excluded from the results because it was cytotoxic

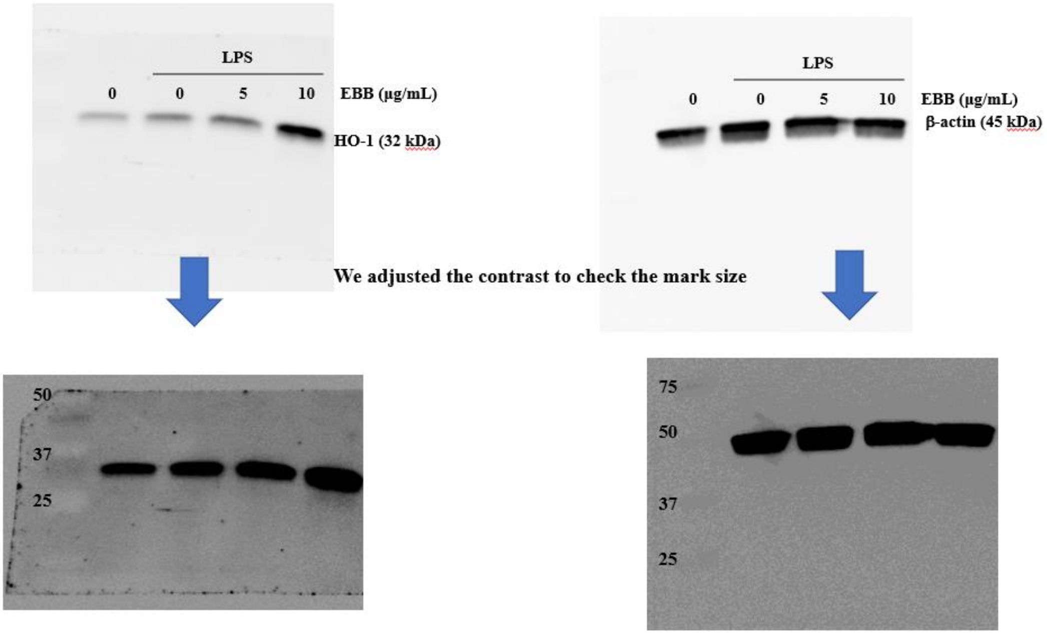

Supplementary Figures S3. Original figure of western blot for Figure 4c. If you adjust the contrast, you can see the marker band. After confirming with that, we checked the band at the desired location.

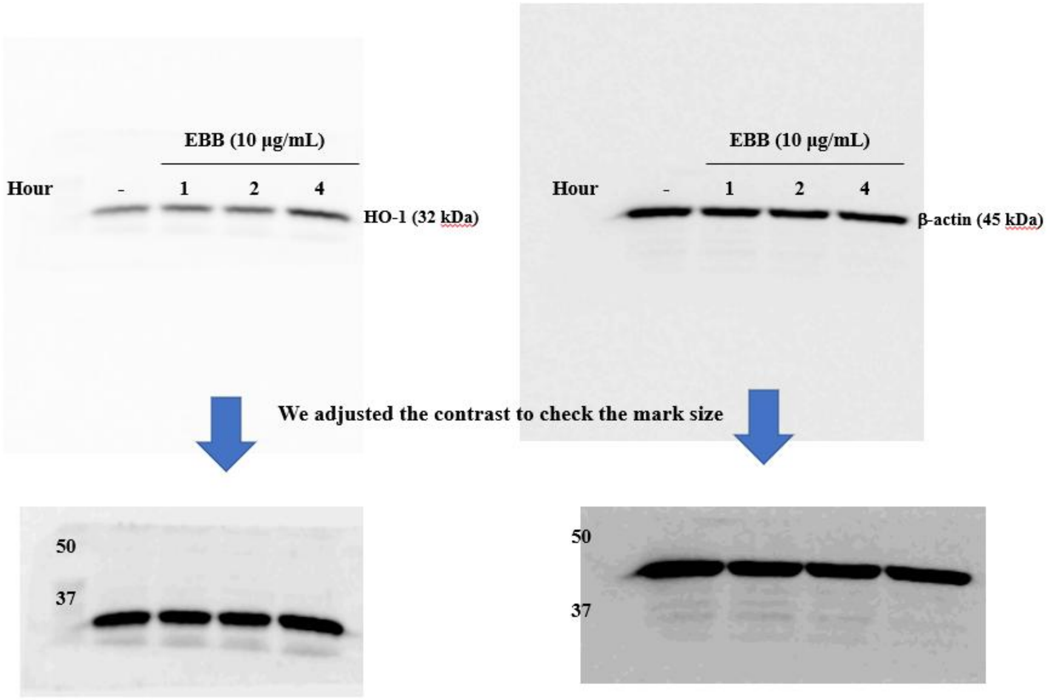

Supplementary Figures S4. Original figure of western blot t for Figure 6a. If you adjust the contrast, you can see the marker band. After confirming with that, we checked the band at the desired location.

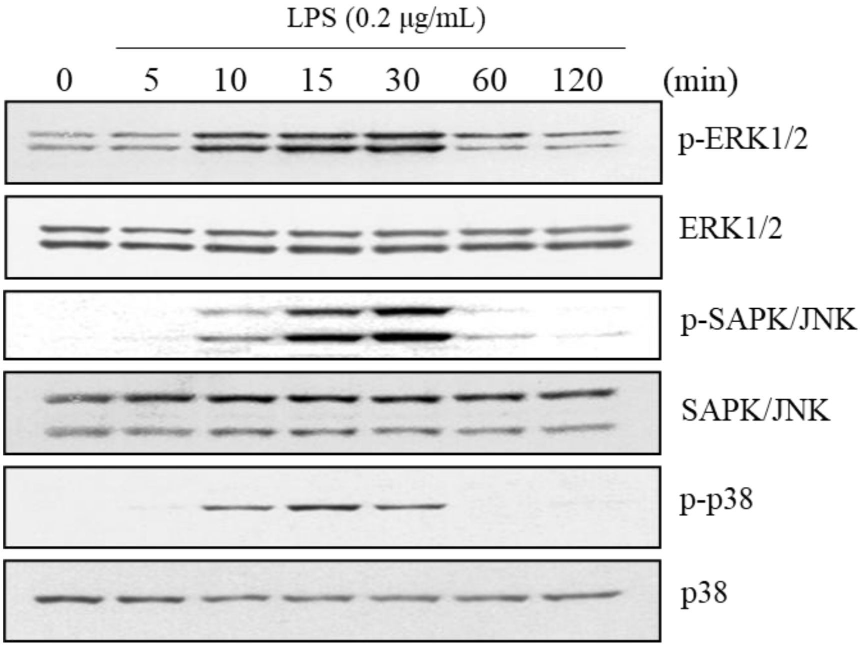

Supplementary Figures S5. Time-course analysis of ERK1/2, SAPK/JNK, and p38/MAPK phosphorylation in response to LPS. BV2 cells were treated with LPS (0.2 μg/mL). Equal amounts of cell lysates, harvested at the indicated time points, were analyzed for ERK1/2, SAPK/JNK, and p38/MAPK phosphorylation by western blot analyses
